# Genetic Variation and Population Genetics of *Taenia saginata* in North and Northeast Thailand in relation to *Taenia asiatica*

**DOI:** 10.1155/2013/310605

**Published:** 2013-06-24

**Authors:** Malinee Anantaphruti, Urusa Thaenkham, Teera Kusolsuk, Wanna Maipanich, Surapol Saguankiat, Somjit Pubampen, Orawan Phuphisut

**Affiliations:** Department of Helminthology, Faculty of Tropical Medicine, Mahidol University, 420/6 Ratchawithi Road, Bangkok 10400, Thailand

## Abstract

*Taenia saginata* is the most common human *Taenia* in Thailand. By *cox1* sequences, 73 isolates from four localities in north and northeast were differentiated into 14 haplotypes, 11 variation sites and haplotype diversity of 0.683. Among 14 haplotypes, haplotype A was the major (52.1%), followed by haplotype B (21.9%). Clustering diagram of Thai and GenBank sequences indicated mixed phylogeny among localities. By MJ analysis, haplotype clustering relationships showed paired-stars-like network, having two main cores surrounded by minor haplotypes. Tajima's *D* values were significantly negative in *T. saginata* world population, suggesting population expansion. Significant Fu's *F*_*s*_ values in Thai, as well as world population, also indicate that population is expanding and may be hitchhiking as part of selective sweep. Haplotype B and its dispersion were only found in populations from Thailand. Haplotype B may evolve and ultimately become an ancestor of future populations in Thailand. Haplotype A seems to be dispersion haplotype, not just in Thailand, but worldwide. High genetic *T. saginata* intraspecies divergence was found, in contrast to its sister species, *T. asiatica*; among 30 samples from seven countries, its haplotype diversity was 0.067, while only 2 haplotypes were revealed. This extremely low intraspecific variation suggests that *T. asiatica* could be an endangered species.

## 1. Introduction

Human taeniasis occurs worldwide. It is caused by *Taenia saginata* and *T. solium*. A third species, *T. asiatica*, is an additional source of intestinal infection in a number of Asian countries. Cattle are the most common source of *T*. *saginata *infection, while the most common cause of both *T. solium *and *T*. *asiatica* infection is swine. *T. solium* metacestodes, called *Cysticercus cellulosae*, reside in the animal's muscle, whereas *T*. *asiatica* metacestodes, called *C. viscerotropica*,  parasitize the liver. In its evolution, speciation of *Taenia* appears to be linked primarily to host switching among carnivore definitive hosts [1]. This association between *Taenia* and humans is thought to have developed about 10,000 years ago, coincidental with the development of agriculture and the domestication of food animals, like cattle and pigs [2]. Contraction of *Taenia* tapeworms by humans happened independently twice, both times by host switching from carnivore definitive hosts to primate definitive hosts [3]. One is an ancestor of *T. saginata + T. asiatica*, and the other is *T. solium.* Geographical distribution has been extensively modified by European exploration and colonization since the 1500s and by ongoing globalization of agriculture and the changing patterns of human migration [4].

Understanding the genetic population structure of parasites helps to elucidate parasite transmission patterns and develop control measures [5]. The population structure and genetic variation of *T. solium* revealed two separate groups: Asian and African/Latin American genotypes [6]. Intraspecies strain variation among *T. solium* has been found to be minimal [7, 8]. Livestock farming of intermediate swine hosts may be reducing the possibility of genetic variation in *T. solium*. On the other hand, cattle, an intermediate host of *T. saginata*, are found in herds in a wide range of pastures. With different farming methods of this intermediate host, the population structure of *T. saginata* requires investigation.

Cytochrome *c* oxidase subunit I (*cox1*) genes of mitochondrial DNA have been commonly used for studying phylogenetic relationships among taeniid cestodes. Distinct intraspecific variations have been detected among various species, for example, *Echinococcus granulosus* [9], *E. multilocularis* [10], and *T. taeniaeformis* [11]. However, little is known about *cox1* genetic variation within a human *Taenia* species. A minor variation was observed in one isolate of *T. saginata* from Kenya and Poland [12], which compared mitochondrial* cox1* and nuclear rDNA 28S sequences. In this study, we focused on the genetic variation of *T. saginata* among samples collected from various geographical localities in north and northeast Thailand, where this parasite is highly prevalent among local inhabitants [13, 14]. In-depth studies of the genetic divergence of *T. saginata* specimens in Thailand have never been conducted. Indeed, the genetic structure among populations of this species and its evolution in Thailand and throughout the world remains limited. Our aim, using partial sequencing of the mitochondrial *cox1* gene, was to examine intraspecific variations and the population genetics of* T. saginata *in Thailand. The genetic divergence of the sister species, *T. asiatica*, from Thailand was also considered.

## 2. Materials and Methods

### 2.1. Studied Host Population and Parasites

**Figure 1:**
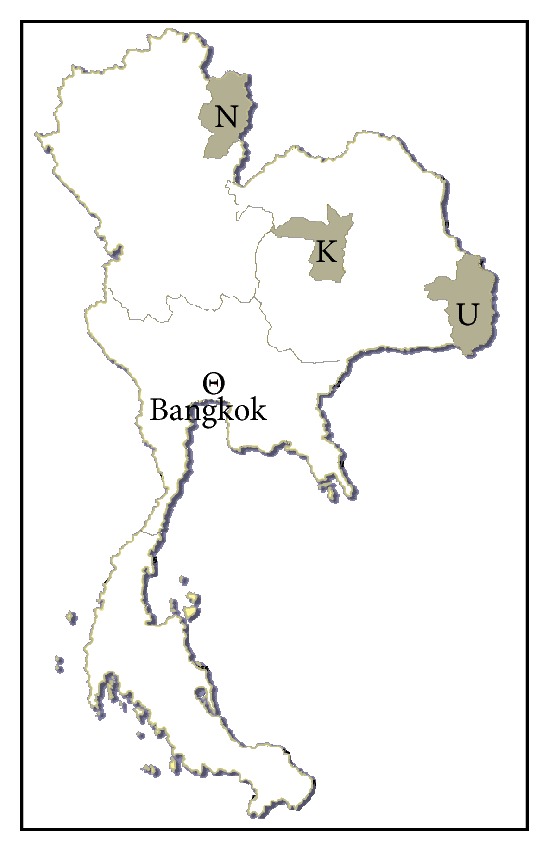
Map of Thailand showing study areas. N, Nan; U, Ubon Ratchathani; K, Khon Kaen.

Parasites were collected during the years 2009-2010 from four sites in north and northeast Thailand. The two sites in the north—one in the lowlands, the other in the upland hill tribe communities—are both in the Thung Chang district, Nan province, an area on the northern border with Lao PDR. The two sites in the northeastern region are in two different provinces: Ubon Ratchathani and Khon Kaen. Ubon Ratchathani shares borders with both southern Lao PDR and northern Cambodia. Khon Kaen lies closer to the center in the upper half of Thailand (Figure 1).

*T. saginata* was collected from both *Taenia* egg-positive persons and from persons who spontaneously discharged gravid proglottids. The worms were identified morphologically as *T. saginata* by scolex and/or gravid proglottids. Male infection rates were almost double females, at a ratio of 43 : 23. The ages of the infected individuals ranged between 12 and 83 years. The worms were fixed in 70% ethanol for molecular analysis. This study was approved by the Ethics Committees of the Faculty of Tropical Medicine, Mahidol University, and the Ministry of Public Health, Thailand. Informed consent was obtained prior to subject participation.

Twelve* T. asiatica*, previously collected from Kanchanaburi Province [15], were coprocessed with *T. saginata*. The *cox1* sequences of 33 *T. saginata* and 18 *T. asiatica* from various different countries in the world, deposited in the GenBank database (Table 1), were also analyzed.

### 2.2. Molecular Studies

#### 2.2.1. DNA Analysis

Partial proglottid fragments of individual strobila were separated and washed with distilled water to remove any ethanol remaining from the fixation process. The genomic DNA of each worm was extracted using a Genomic DNA Mini Kit (Geneaid, Sijhih City, Taiwan) per the manufacturer's instructions. DNA was resuspended in 50 *μ*L elution buffer (provided as part of the kit). The PCR amplicons were amplified using two oligonucleotide primers: *cox1* (forward), 5′-CATGGAATAATAATGATTTTC-3′, and *cox1* (reverse), 5′-ACAGTACACACAATTTTAAC-3′. These primers were designed from the alignment of *T. saginata *and* T. asiatica *mitochondrial *cox1* genes (AB533171 and AB533175, resp.). PCR amplicons were produced in 50 *μ*L of reaction mixture, containing: 10 ng genomic DNA; 0.5 *µ*M of each primer; and 1x TopTaq Master Mix Kit (comprising TopTaq DNA Polymerase, PCR Buffer with 1.5 mM MgCl_2_, and 200 *μ*M each dNTP) (QIAGEN, Germany). Amplification conditions were as follows: initial heating at 94°C for 3 min, followed by 30 amplification cycles, consisting of denaturation at 95°C for 30 sec, annealing at 53°C for 30 sec, and elongation at 72°C for 50 sec. PCR products were run into 1.2% agarose gel and visualized with a UV illuminator. The PCR amplicons were purified and sequenced by dideoxytermination method, using an ABI3730XL sequencer and BigDye v 3.1 (Applied Biosystems, Foster City, CA, USA) at Macrogen Inc. (Geumcheon-gu, Seoul, Republic of Korea). DNA sequences were aligned using the BioEdit program, version 7.0 [16]. There is no conflict of interests with the commercial identities in this paper.

#### 2.2.2. Population Genetic Analysis

The 924 bp *cox1* gene population genetics of *T. saginata* samples from four different localities was analyzed. Genetic diversity values, including polymorphic sites between populations (*S*), haplotype numbers (*h*), haplotype diversity (*Hd*), nucleotide diversity (*π*), theta-*w * (*θw*), and theta-*π* (*θπ*) estimators to measure DNA polymorphism [17–19], were calculated using DnaSP version 4.0 [20] and the Arlequin computer program, version 3.1 [21, 22]. These programs were also used to evaluate the genetic structure of the parasites under the population expansion effect, via Tajima's *D* test and Fu's *F*_*s*_ test [23].

#### 2.2.3. Clustering Diagram and Haplotype Network Analysis

The *cox1* sequences were aligned by ClustalX version 2.0 [24], and the haplotypes then distinguished. A neighbor-joining (NJ) phylogram was constructed under *p*-distance model by MEGA version 5.0 [25]. Bootstrap analyses were conducted using 1,000 replicates. A median-joining (MJ) network of *cox1* haplotypes was illustrated by Network 4.5.1.6 Software (Fluxus Technology Ltd. (http://www.fluxus-engineering.com/)). The *T. saginata* world populations (Table 1) were also tested for genetic differentiation without regional separation by global AMOVA.

## 3. Results

### 3.1. Parasites and Infections

A total of 73 *Taenia saginata* isolates were collected from 66 cases across the four study sites in north (Nan lowland, NL; Nan highland, NH) and northeast (Ubon Ratchathani, UB; Khon Kaen, KK) Thailand (Figure 1). The samples studied were 16 and 32 isolates from lowland and highland Nan Province in the north, and 9 and 16 isolates from Ubon Ratchathani and Khon Kaen provinces, in the northeast.

### 3.2. Mitochondrial *cox1* DNA Sequence Analyses

Total DNA was extracted from 73 *Taenia* samples from four different geographical localities and then processed for sequencing. The partial cytochrome *c* oxidase subunit 1 (*cox1*) sequences confirmed that they were all *T. saginata* (GenBank accession nos. JN986646 to JN986718). The 924 bp *cox1* sequences of these samples were divided into 14 discrete groups, represented as haplotypes A–N, and revealed 11 segregation (polymorphic) sites (*S*). Percentage intraspecific variation was 1.2%, with 1–5 nucleotide substitutions (Tables 2 and 3). Among these diverse haplotypes, two main ones had the highest ratio. Haplotype A was the most dominant haplotype (38/73, 52.1% of samples), in total and across all four localities. Haplotype B was the second most dominant (16/73 isolates, 21.9%) and was also detected in all localities. Between the two main haplotypes, A and B, there were two nucleotide substitutions (0.2%). The other haplotypes (haplotypes C–N) were detected in only 1–3 isolates (Table 2).

### 3.3. Population Genetics

The genetic diversity value of the 73 samples taken from the four localities, determined by haplotype diversity (*Hd*), was 0.683 ± 0.05; the nucleotide diversity (*π*) was 0.00146 ± 0.00017.  *θw* was greater than *θπ*. Tajima's *D* test of neutrality showed no significant value (−1.102, *P* = 0.112) in these samples. A significant Fu's *F*_*s*_ value was, however, revealed (−7.565, *P* = 0.001) (Table 3). The *cox1* gene sequences of 33 *T. saginata* in different geographical areas from the GenBank database (Table 1) were included in the analysis. From 106 samples, 23 haplotypes (*h*) and 23 polymorphic sites (*S*) were revealed. Entire intraspecific variation was 2.5%. Mean haplotype diversity (*Hd*) was 0.686, and mean intrapopulation nucleotide diversity (*π*) was 0.00155. *θw* was greater than *θπ* (Table 3). The nucleotide sequence of Haplotype A in our study (38) was identical to *T. saginata* in GenBank data for Thailand(7) and the other countries analyzed (China(2), Korea(1), Japan(1), Indonesia(2), Nepal(1), Ecuador(1), Brazil(2), Ethiopia(1), and Belgium(1)). The nucleotide sequence of Haplotype B was unique among the Thailand isolates, that is, 16 samples in this study and 3 from GenBank. The frequency of Haplotype A was 53.8% (57/106 samples); the frequency of Haplotype B was 17.9% (19 from 106 samples). Our sample sequences of Haplotype C–N and the GenBank sequences of Haplotype O–Q were unique among the Thailand isolates (Table 2). Some samples from China, Cambodia, Ecuador, Brazil, and Ethiopia showed nonidentical sequences (Haplotype R–W) (Table 2). In the GenBank data samples analyzed, noteworthy Tajima's* D* value and Fu's *F*_*s*_ value (−1.878, −18.798) were observed (Table 3).

In looking at the sister species, *T. asiatica*, significant differences between* T. asiatica* and *T. saginata* were discovered. The 924 bp *cox1* gene sequences of the 12 isolates from Kanchanaburi Province, Thailand, were all identical. Among 30 samples from seven different countries—China (4), Taiwan (1), Korea (2), Japan (3), the Philippines (1), Indonesia (3), and Thailand (16)—only two haplotypes of the *cox1* gene were found, where the major haplotype comprised 29 samples. Only one sample from China had other haplotypes, and only one polymorphic site was found. Haplotype diversity *Hd* was 0.067. However, the value of nucleotide diversity *π* of each of the two species was very low (Table 3).

### 3.4. Clustering Diagram and Haplotype Network

**Figure 2:**
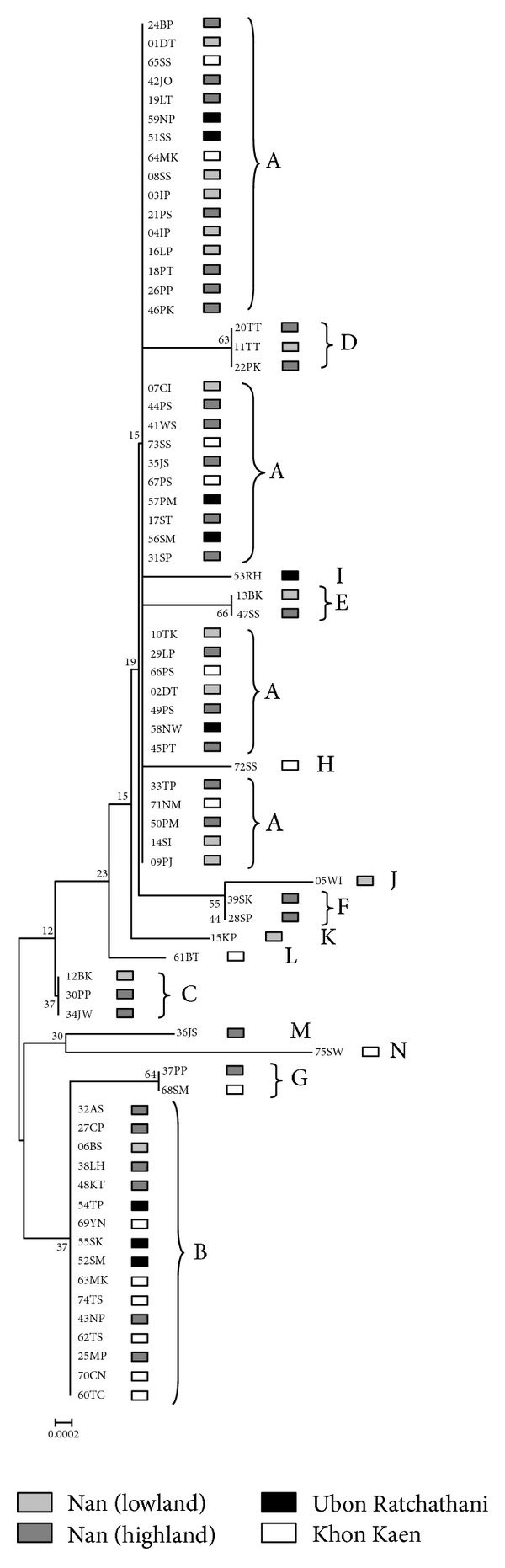
A neighbor-joining phylogram of mitochondrial *cox1*  gene of 73 *T. saginata* from 4 localities of Thailand. A to N indicates haplotype.

**Figure 3:**
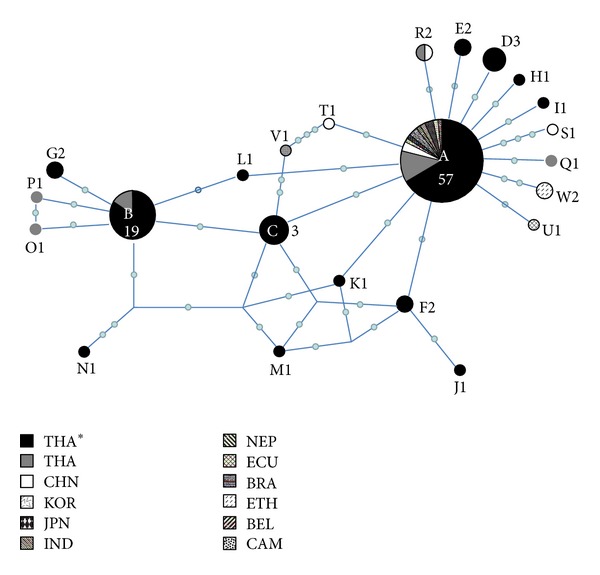
A median-joining network of *T. saginata* from Thailand (THA*, *n* = 73) and other 11 countries (*n* = 33). Haplotype codes (A-W are shown inside/adjacent to the circles. The size of circle denotes that a haplotype is proportional to the number of isolates of each haplotype shown inside/adjacent to the circle. Small circles indicate the number of nucleotide substitutions. THA: Thailand, CHN: China, KOR: Korea, JPN: Japan, IND: Indonesia, NEP: Nepal, ECU: Ecuador, BRA: Brazil, ETH: Ethiopia, BEL: Belgium, and CAM: Cambodia.

The clustering diagram of *T. saginata cox1* gene indicated no significant genetic differentiation among the populations from the four different localities studied. *T. saginata* could not be separated by geographical locality, that is, upper north, east of, northeast, and central northeast Thailand (Figure 2). Similarly, *T. saginata* from the different countries (see Table 1) was not geographically discriminated. The median-joining (MJ) clustering relationship was constructed as a haplotype network (Figure 3). All haplotypes appeared to be separated into two groups which each cored at the two main haplotypes, A and B. Connectivity between them was seen with a number of shared divergent haplotypes. In addition, unique sequences of samples from different geographical countries, Haplotypes Q–W, showed connectivity to Haplotype A. Among these haplotypes, 1–4 nucleotide substitutions were revealed (Table 2). Additionally, of samples from Thailand only in the GenBank database, three had Haplotype B sequences; two unique sequence haplotypes (O and P) were connected to Haplotype B (Figure 3).

## 4. Discussion

In this study, we investigated the population variation of *Taenia saginata *in Thailand. The results showed 11 haplotypes where distribution was not related to geographical locality. Samples in our study from the north (*n* = 48), northeast (*n* = 25), and west (*n* = 8) (AB465231-3, AB465242, AB465247-8, AB465236, and AB465239) of Thailand were mixed together. Intraspecific variations in *cox1* genes have been reported among *T. saginata *samples from a number of different localities, including China, Ethiopia, France, Indonesia, Korea, Lao PDR, the Philippines, Taiwan, Thailand, and Switzerland. Global genetic divergence was 1.2–1.8% in the nucleotide variant positions of the total 1620 bp length [26, 27]. In terms of nucleotide divergence of large-scale samples from Thailand, 11*S*, 1.2% displayed the results of isolates from global infection. However, among the worldwide* T. saginata* populations (*n* = 106), the Thai isolates (*n* = 73) and GenBank isolates (*n* = 33) used in this analysis, a high level of genetic variation (23*S*, 2.5%) was found. The genetic diversity values, haplotype diversity (*Hd*), and nucleotide diversity (*π*) found in populations in this study and in other combined populations were similar.

By MJ network, Haplotype B showed connectivity to Haplotype A in the *T. saginata* world population. In the *T. saginata* sequences, 19 of 33 (57.6%) from GenBank were identical to Haplotype A. Consequently, the star-like expansion in the MJ network of the major haplotype confirms Haplotype A as an ancestor among the *T. saginata* world population. It also suggests that the subpopulation of minor haplotypes recently experienced a significant increase from its ancestors. Haplotype B and its star-like expansion network were unique to the Thailand isolates. Indeed, it is possible for Haplotype B diverged genetically to be the recent common ancestor of *T. saginata* in Thailand. However, the major Haplotype A contained half (*n* = 57) of the total population we analyzed. This means that *T. saginata* Haplotype A may share its genetic ancestry with populations from a variety of different geographical areas, in Asia as well as those in other continents. *θπ* less than *θw* indicates purifying selection, which results in selective removal of the deleterious allele in the population [18]. Tajima's *D* value is significantly negative in the world population but not in the Thailand population. The significant Fu's *F*_*s*_ value revealed in isolates from both Thailand and the world population, however, suggests that the population is growing and is hitchhiking due to population expansion and selective sweep [23]. The samples analyzed from each country were too small to be able to estimate this expansion and the genetic structure of *T. saginata* populations worldwide.

*T. asiatica* is the third *Taenia* tapeworm of humans and is reported only in Asian countries. It is distributed in specific areas across several countries, including Taiwan [28], Korea [29], China [30], Vietnam [31], Indonesia [32], and Thailand [33]. It is estimated that *T. saginata* and *T. asiatica* diverged from other human tapeworms about one million years ago (Mya), 0.414–1.616 Mya. *T. saginata* lineages emerged at an earlier period than *T. asiatica*. Lineages of *T. saginata* emerged at 238,000 years, while that of *T. asiatica *were at 41,000 years [34]. Very low intraspecies diversity of *T. asiatica* has been observed. Identical partial *cox1* gene nucleotide sequences have been found in 5 isolates (366 bp) from unspecified areas of Taiwan [12]; 17 isolates (337 bp) have been found across different localities in Korea [27]; and 12 isolates (924 bp) have been found in Kanchanaburi Province of Thailand. Of the total 1620 bp sequence length, only two variant nucleotide positions (0.1%) were detected in 5 isolates from China (2), the Philippines (1), and Korea (2) [26]. The low genetic variation of *T. asiatica* suggests populations of *T. asiatica* tapeworm to be small. The prevalence of *T. asiatica* is low when compared to *T. saginata* infections in most countries [35–37]. Also, Tajima's *D* value revealed no gene flow in *T. asiatica*, a result which indicates obvious differences in the population structures of *T. saginata* and *T. asiatica*. To date, since the emergence of *T. saginata* and *T. asiatica*, cattle have been known to be the intermediate hosts for *T. saginata* and swine for *T. asiatica*. The livestock management of these two intermediate host species has been different, and this may be an impact factor for these parasites. Pigs are raised feeding in restricted shed areas; gene flow among parasite populations in pigs, therefore, is diminished. In cattle farming, especially in Thailand, the animals are herded by grazing on naturally grown pastures, particularly pastures of postharvest rice which often cover wide distances. Furthermore, such cattle are frequently untransported, while daily herds are moved to a main city slaughterhouse over a relatively long period of time. The chances of cattle coming into contact with contaminated *Taenia* eggs, whether from human carriers during grazing or whilst drinking stream water from place to place, remain. This supports no specific locality in *T. saginata* populations for each genotype. It may even suggest that *T. saginata* tapeworm populations migrated during host cattle farming. Gene flow among *T. saginata* may have been influenced by host population migration. The difference in population genetics found in *T. saginata* suggests that intraspecies populations are growing. On the other hand, its sister species, *T. asiatica,* reveals very low genetic diversity. Such low divergence may indicate a loss of potential adaptive alleles for surviving in a changing environment, which could lead to the overall reduction of *T. asiatica* populations.

Cattle also act as definitive hosts for the liver fluke *Fasciola* spp. Ichikawa et al. [38] investigated the 535 bp partial nucleotide sequences of the *nad1* gene in 88 adult *Fasciola* flukes from three localities in Myanmar and found 27 substitution sites that yielded 20 haplotypes. A major haplotype revealed 54.6% (48/88 flukes) frequency and was seen in all three areas regardless of locality. The intraspecies genetic variation in* Fasciola* spp. is thought to have been introduced to Myanmar through ancient anthropogenetic movements of domestic ruminants. This seems to be the main factor determining mixing of the parasite population. Likewise, cattle host movements suggest intraspecific genetic variations of *T. saginata* populations in our study. Despite the fact that cattle serve as the definitive host for *Fasciola* spp. but the intermediate hosts for *T. saginata*, this status of intermediate host and definitive host does not influence different genetic variation of parasite species.

Our work shows that *T. saginata* adult worm isolates from humans, from two locations in the north and two provinces in northeast Thailand, exploited intraspecific genetic variability, without correlation with the geographical region of origin. The phylogenetic network of* cox1* sequences revealed 14 haplotypes from 73 samples. Thirty-three sequences from GenBank were added, and 23 haplotypes were exploited among the 106 samples. The genetic divergence of world *T. saginata* populations was 2.5%. Two main haplotypes, A and B, showed connectivity between them. Haplotype A seems to be an ancestor of *T. saginata* in the world population. Haplotype B and its dispersion are unique to the Thailand population. Intensive studies and a greater number of samples from different geographical areas are required to clarify the population genetics of *T. saginata* both in Thailand and worldwide.

## Figures and Tables

**Table 1:** Accession numbers of 73 *T. saginata  cox1* sequences in this study, 33 from 11 different geographical countries, and 30 *T. asiatica *isolates from 7 different countries deposited in GenBank.

Species	Number of samples	Locality (country)	Accession numbers
*T. saginata *	5	China	AB107239, AB107247, AB533168, AB533169, and AB533171
	1	Korea	AB465246
	1	Japan	AB465244
	2	Indonesia	AB107240, AB465240
	1	Cambodia	AB465241
	1	Nepal	AB107243
	2	Ecuador	AB107238, AB465243
	3	Brazil	AB107237, AB107246, and AB465238
	3	Ethiopia	AB107241, AB465237, and AB465245
	1	Belgium	AB107242
	13	Thailand	AB107244, AB107245, AB465231, AB465232, AB465233, AB465234, AB465235, AB465236, AB465239, AB465242, AB465247, AB465248, and AB533173
	73	Thailand, this study	JN986646–JN986718
*T. asiatica *	4	China	AB465211, AB465212, AB465213, and AB465227
	1	Taiwan	AB465230
	2	Korea	AB465224, AB465225
	3	Japan	AB608736, AB608739, and AB608742
	1	Philippines	AB465229
	3	Indonesia	AB465215, AB465216, and AB465228
	4	Thailand	AB533174, AB533175, AB465222, and AB465223
	12	Thailand, this study	JQ517298–JQ517309

**Table 2:** Haplotype, nucleotide variation sites of partial *cox1* gene (924 bp length), and frequency of 73 *Taenia saginata* samples in this study (THA*, haplotype A–N) and 33 from 11 different geographical countries** (Haplotype O–W).

Number	Haplotype	Position of nucleotide change																							Number of individuals per population																
		0	0	1	1	2	2	2	3	3	4	5	6	6	6	7	7	7	7	8	8	9	9	9	NL	NH	UB	KK	Subtotal THA*	THA	CHN	KOR	JPN	IND	CAM	NEP	ECU	BRA	ETH	BEL	Total
		3	6	7	8	1	3	3	0	8	9	7	2	3	8	1	2	6	9	0	9	0	1	2																	
		9	3	4	6	9	1	7	0	1	2	0	1	9	7	1	3	9	8	6	0	1	2	4																	
1	A	C	G	G	A	C	A	C	T	A	A	A	G	A	C	T	T	A	T	C	A	C	G	T	10	17	5	6	38	7	2	1	1	2	—	1	1	2	1	1	57
2	B	.	·	·	·	·	·	·	·	·	·	·	·	·	T	·	·	·	·	·	·	T	·	·	1	6	3	6	16	3	—	—	—	—	—	—	—	—	—	—	19
3	C	·	·	·	·	·	·	·	·	·	·	·	·	·	·	·	·	·	·	·	·	T	·	·	1	2	—	—	3	—	—	—	—	—	—	—	—	—	—	—	3
4	D	·	·	·	G	·	·	·	·	·	·	·	·	·	·	·	·	·	·	·	·	·	·	·	1	2	—	—	3	—	—	—	—	—	—	—	—	—	—	—	3
5	E	·	·	·	·	·	·	·	·	·	·	·	·	G	·	·	·	·	·	·	·	·	·	·	1	1	—	—	2	—	—	—	—	—	—	—	—	—	—	—	2
6	F	·	·	·	·	·	·	·	·	G	·	·	·	·	·	·	·	·	·	·	·	·	·	·	—	2	—	—	2	—	—	—	—	—	—	—	—	—	—	—	2
7	G	·	·	·	·	T	·	·	·	·	·	·	·	·	T	·	·	·	·	·	·	T	·	·	—	1	—	1	2	—	—	—	—	—	—	—	—	—	—	—	2
8	H	·	·	·	·	·	·	·	·	·	·	·	·	·	·	C	·	·	·	·	·	·	·	·	—	—	—	1	1	—	—	—	—	—	—	—	—	—	—	—	1
9	I	·	·	·	·	·	·	·	·	·	·	·	·	·	·	·	·	T	·	·	·	·	·	·	—	—	1	—	1	—	—	—	—	—	—	—	—	—	—	—	1
10	J	·	·	·	·	·	·	·	·	G	·	·	·	·	·	·	·	·	C	·	·	·	·	·	1	—	—	—	1	—	—	—	—	—	—	—	—	—	—	—	1
11	K	·	·	·	·	·	·	·	·	·	·	·	·	·	·	·	·	·	·	·	C	·	·	·	1	—	—	—	1	—	—	—	—	—	—	—	—	—	—	—	1
12	L	·	·	·	·	·	·	·	·	·	·	·	·	·	T	·	·	·	·	·	·	·	·	·	—	—	—	1	1	—	—	—	—	—	—	—	—	—	—	—	1
13	M	·	·	·	·	·	·	·	·	G	·	·	·	·	·	·	·	·	·	·	C	T	·	·	—	1	—	—	1	—	—	—	—	—	—	—	—	—	—	—	1
14	N	·	·	·	·	·	·	·	·	·	·	T	·	·	T	·	·	·	A	·	C	T	·	·	—	—	—	1	1	—	—	—	—	—	—	—	—	—	—	—	1
15	O	·	·	·	·	·	·	·	·	·	·	·	·	·	T	·	·	·	·	·	·	T	·	C						1	—	—	—	—	—	—	—	—	—	—	1
16	P	·	·	·	·	·	·	·	·	·	·	·	·	·	T	·	·	·	·	·	·	T	·	A						1	—	—	—	—	—	—	—	—	—	—	1
17	Q	·	·	·	·	·	·	·	·	·	G	·	·	·	·	·	·	·	·	·	·	·	·	·						1	—	—	—	—	—	—	—	—	—	—	1
18	R	·	A	·	·	·	·	·	·	·	·	·	·	·	·	·	·	·	·	·	·	·	·	·						—	1	—	—	—	1	—	—	—	—	—	2
19	S	·	·	A	·	·	G	·	·	·	·	·	A	·	·	·	·	·	·	·	·	·	·	·						—	1	—	—	—	—	—	—	—	—	—	1
20	T	·	·	·	·	·	·	T	·	·	·	·	·	·	·	·	·	·	·	·	·	·	·	·						—	1	—	—	—	—	—	—	—	—	—	1
21	U	·	·	·	·	·	·	·	·	·	·	·	·	·	·	·	·	·	·	T	·	·	·	·						—	—	—	—	—	—	—	1	—	—	—	1
22	V	T	·	·	·	·	·	T	·	·	·	·	·	·	·	·	C	·	·	·	·	T	·	·						—	—	—	—	—	—	—	—	1	—	—	1
23	W	·	·	·	·	·	·	·	C	·	·	·	·	·	·	·	·	·	·	·	·	·	A	·						—	—	—	—	—	—	—	—	—	2	—	2

Dots reprepresent homology with haplotype A sequence.

NL: Nan lowland, NH: Nan highland, UB: Ubon Ratchathani, KK: Khon Kaen.

**11 countries including THA: Thailand, CHN: China, KOR: Korea, JPN: Japan, IND: Indonesia, CAM: Cambodia, NEP: Nepal, ECU: Ecuador, BRA: Brazil, ETH: Ethiopia, and BEL: Belgium.

The numbering of nucleotide position of 1–924 referred to position 400–1324 of the complete mtDNA sequence (1620 bp) of *T. saginata* (GenBank acc.  no.  AB066495).

**Table 3:** Genetic diversity and test of *T. saginata* population, 73 from 4 different geographical regions of Thailand, 33 from 11 different countries and of *T.  asiatica*, 12 from Kanchanaburi province, and 18 from 7 different countries.

Species	Population	Number of samples	*h*	*S* (%)	Genetic diversity				Neutrality tests	
					*Hd*	*π*	Theta-*w*	Theta-*π*	Tajima's *D* (*P* value)	Fu's *F*_*s*_ (*P* value)
*T. saginata *	Thai population	73	14	11 (1.2)	0.683 ± 0.050	0.00146 ± 0.000	2.263 ± 0.875	1.350 ± 0.093	−1.102 (0.112)	−7.565* (0.001)
	World population	106	23	23 (2.5)	0.686 ± 0.045	0.0155 ± 0.0001	4.194 ± 1.319	1.487 ± 1.002	−1.878* (0.004)	−18.798* (0.000)
*T. asiatica *	Thai population	12	0	0	0	0	ND	ND	ND	ND
	Asian population	30	2	1	0.067	0.000	ND	ND	−1.15	ND

*h*: haplotype numbers, *S*: number of segregation sites, *Hd*: haplotype diversity, *π*: nucleotide diversity, Theta-*w*: Watterson's theta based on *S*, Theta-*π*: the theta based on *π*.  *Significance (*P* < 0.05).
